# The critique of psychiatry as we enter the third decade of the 21st century: Commentary on… Critical psychiatry

**DOI:** 10.1192/bjb.2020.10

**Published:** 2020-12

**Authors:** Mohammed Abouelleil Rashed

**Affiliations:** Department of Philosophy, School of Social Sciences, History and Philosophy, Birkbeck College, University of London, UK

**Keywords:** Critical psychiatry, biopsychosocial model, reductionism, mental health activism, concept of mental disorder

## Abstract

Critical psychiatry takes the position that ‘mental illness’ should not be reduced to ‘brain disease’. Here I consider whether this particular stance is outdated in light of more recent exchanges on reductionism, which consider questions raised by new mental health sciences that seek truly integrative and specific biopsychosocial models of illness.

## The anti-reductionist position of critical psychiatry

According to Duncan Double, the essential position of critical psychiatry is that ‘functional mental illness should not be reduced to brain disease’.^1^ As it stands, this claim is ambiguous with regard to the basis of the normative (ethical) prescription contained within it: why does critical psychiatry maintain that we should not reduce ‘mental illness’ to ‘brain disease’? We are provided with a clue as to Double's view when he writes that ‘most psychopathology is functional, in the sense that there are no structural abnormalities in the brain’.^1^ Accordingly, we should not engage in such a reduction because we do not possess the evidence that it can be done. To insist on this reduction, and on the treatments and practices it entails, can therefore be construed as ethically problematic. Now this sort of justification for critical psychiatry's essential position lands us in indeterminate territory: it relies on establishing the presence of ‘structural abnormalities’, which depends on how we define this term, on the state of the science and on the nature of evidence and its interpretation. There will always be claims and counterclaims as to the evidence for the biological basis of ‘mental illness’.

There is another more substantial argument that can be provided in support of critical psychiatry's position: we should not engage in the offending reduction because even if there were ‘structural abnormalities in the brain’, the subject matter – ‘mental illness’ – requires an altogether different approach for its classification, understanding and management. Double seems to intend something like this when he argues, along with von Feuchtersleben, Meyer and Engels, for a holistic, non-reductionist and integrative conception of ‘psychopathology’. This is a valuable analysis, and it is hard to disagree with the general direction of Double's argument in this regard: mental health conditions cannot be understood, treated or managed through radically reductionist concepts and approaches. And radical reductionism is the villain that critical psychiatry, on Double's account, is engaged in a fight with. It is a reductionism that is so obtuse, it has no place for meaning, human relationships or social context. And it was certainly evident in the early days of psychiatry, through the wild goose chase prompted, in part, by the discovery of the celebrated causal connection between general paresis of the insane and the syphilis spirochaete. How far does this view exist today?

We can quarrel endlessly about critical psychiatry's representation of the views of its opponents, and it is unlikely that we can ever resolve this to everyone's satisfaction. Instead, I shall introduce a more recent exchange on reductionism, and from there we can discern where things are at and what critical psychiatry has to say to us today.

## Towards a truly integrated understanding of health

In a paper published in the *BMJ* in 2012, White and colleagues argue for an end to the distinction between mental disorders and brain disorders in favour of a single overarching category: disorders of the nervous system.^2^ One of the arguments they make in support of this proposal is that psychosocial factors ‘interact strongly’ with neurological disorders, whereas ‘disorders of the mind are rooted in dysfunction of the brain’.^2^^,^^3^ Psychological, social and biological causal and risk factors run across all medical conditions, whether mental or physical.^2^ If so, there might not be much sense insisting on the distinction between mental disorders and brain disorders, especially, they argue, considering recent advances in the neurological and genetic bases of mental disorders. Standing in contrast to White and colleagues’ proposal is a position paper by Bracken and colleagues that rejects the suggested equivalence between psychiatry and neurology: ‘Psychiatry is not neurology; it is not a medicine of the brain. Although mental health problems undoubtedly have a biological dimension, in their very nature they reach beyond the brain to involve social, cultural and psychological dimensions’.^4^

These two papers, although they argue for opposite conclusions, are both non-reductionist in the sense that they do not propose a radical disavowal of non-biological causal and risk factors. Accordingly, neither falls foul of critical psychiatry's essential position as articulated by Double. A key point on which they differ is a question of emphasis: White and colleagues emphasise genetic and neurological factors, whereas Bracken and colleagues emphasise psychosocial factors. Where should we stand on this point?

There is no doubt that mental health conditions and neurological conditions demonstrate ‘multifactorial pathways’, as White and Colleagues note. Nevertheless, psychosocial factors are more prominent in mental disorders across a wide range of dimensions.^5^ To the extent that this is the case, psychiatry differs from neurology in that it ‘has particular expertise in the management of psychosocial factors as well as internal biological factors’.^5^ We could take this observation to support the view that, in order to preserve the emphasis on psychosocial factors, we should not collapse the distinction between mental disorders and brain disorders. Or we could decide that the terms of the debate and the forced choice between them are out of keeping with developments in the new mental health sciences. Here, gene–environment interactions, social determinants of health over the life-course, individual psychology and neuroscience are all relevant for an integrated understanding of health. As Derek Bolton puts it: ‘[These] new sciences do not work with ideological battles between the biological, the psychological, and the social, the old parallel universes with poor communication between them; rather they work with all of these factors and the diversity of interplay between them’.^5^

There is much work to be done, both empirical and conceptual, to understand how social, psychological and biological factors interact in specific conditions. However, that work applies across the board to all conditions of health-related interest.^6,7^ The aim is to move beyond a biopsychosocial model that is integrative only in name and towards one that can provide concrete risk and causal pathways across the range of factors of relevance to a particular health condition.

## Critical psychiatry may have run its course, but the critique of psychiatry continues

Where does this leave critical psychiatry? Double's editorial seeks to advance two aims. On one hand, it wants to affirm critical psychiatry's continuing relevance as the defender of the position that ‘functional mental illness should not be reduced to brain disease’. On the other hand, it acknowledges that it might be beneficial ‘to look for the continuities, rather than discontinuities, with orthodox psychiatry’.^1^ On the first aim, we have seen that it is possible to debate whether we should end the distinction between mental disorders and brain disorders without falling into the radical reductionism that critical psychiatry positions itself against. Moreover, as suggested above, the new mental health sciences have moved beyond the terms of this debate and seek genuinely integrative and specific biopsychosocial models of health conditions. Accordingly, in so far as critical psychiatry requires the continuing relevance of its essential position, then it might have run its course. This leaves us with the second aim of the editorial, from which, on Double's own analysis, one gets the sense that there is very little that separates critical psychiatry from ‘orthodox psychiatry’. And that is not a bad thing, for it can be taken by critical psychiatry as a triumph, as evidence that its message has got through.

But critical psychiatry does not exhaust the constructive critique of psychiatry and society, which, as we enter the third decade of the 21st century, is going strong. For example, there are continuing debates on the boundaries of illness and on the definition of mental disorder (e.g.^8,9^); there are attempts to resolve the classificatory complexity of mental health conditions and to critique the validity of existing classifications (e.g.^10–12^); questions continue to be raised about the nature of mental disorders (e.g.^13,14^); controversies remain surrounding the efficacy and risks of antidepressant and antipsychotic medications (e.g.^15^); debates continue on the ethical complexities raised by capacity assessments and coercive interventions (e.g.^16,17^); and challenges to medical concepts and approaches – to medicalisation more generally – are experiencing a resurgence through mental health activism (e.g.^18–22^).

In the midst of these exciting and still largely unresolved problems, the version of critical psychiatry presented by Double in his editorial is of historical value; it reminds us of a radically reductionist position that now – in light of developments in the science and philosophy of mental health – appears false and outdated.

## About the author

**Mohammed Abouelleil Rashed** is a Wellcome Trust Institutional Strategic Support Fund Research Fellow in the Department of Philosophy at Birkbeck College, University of London, UK; a Visiting Lecturer in the Department of Philosophy, King's College London; and a medical doctor with postgraduate training in psychiatry.
